# The complete chloroplast genome of *Acer tutcheri* Duthie (Acereae, Sapindaceae): an ornamental tree endemic to China

**DOI:** 10.1080/23802359.2020.1787899

**Published:** 2020-07-07

**Authors:** Zhaowan Shi, Bing Sun, Nancai Pei, Xin Shi

**Affiliations:** Research Institute of Tropical Forestry, Chinese Academy of Forestry, Guangzhou, China

**Keywords:** *Acer tutcheri*, chloroplast genome, ornamental tree, phylogenetic analysis

## Abstract

*Acer tutcheri* Duthie is a popular ornamental tree with reddish leaves and bright red fruits. In this study, we report the complete chloroplast genome of it. The cp genome was determined to be 156,973 bp in length, containing a large single copy (LSC) region of 85,356 bp, a small single copy (SSC) region of 18,111 bp and two separated inverted region of 26,753 bp, respectively. It encodes a total of 132 unique genes, including 87 protein-coding genes, 37 tRNA genes and eight rRNA genes. The phylogenetic analysis indicates that *A. tutcheri* is sister to *A. wilsonii* Rehd.

The genus *Acer* L. (Acereae, Sapindaceae) contains about 129 species of trees and woody shrubs, widely distributed in the northern hemisphere (Xu et al. 2008; Harris et al. 2017). Most of the *Acer* species were planted as ornamental plants all over the World, which leads to their high commercial values (Schmitzer et al. 2009). *Acer tutcheri* Duthie is one of the popular decorative plant with reddish leaves and red samara. This Chinese endemic species is suitable for planted in park and urban spaces in South China. In the present study, we sequenced and assembled the complete chloroplast genome of *A. tutcheri* to provide significant information for the further studies of its genetic diversity and phylogenetic position in the future.

The fresh leaves were collected from a seedling of *A. tutcheri* cultivated in Research Institute of Tropical Forestry, Chinese Academy of Forestry, which was introduced from Nankun Mountain (23°48′10″N, 113°52′48″E), Guangdong Province, China. The voucher specimens (accession number: NKS-31-3) were deposited at the herbarium of South China Botanical Garden (IBSC). The total genomic DNA was extracted from the silica-gel dried leaves following the modified CTAB method (Doyle and Doyle 1987). Genomic library (paired-end, PE = 150 bp) was sequenced on a BGISEQ-500 platform at Beijing Genomics Institute (Shenzhen, China). In total, 2 Gb raw reads were obtained and then filtered by the program Trimmomatic v.0.33 (Bolger et al. 2014). Chloroplast genome assembly was executed on NOVOPlasty 2.6.3 (Dierckxsens et al. 2017), with manual adjustment and annotation using Geneious version 11.0.3 (Kearse et al. 2012). The annotated cp genome of *A. morrisonense* (GenBank accession number: NC_029371) was used as reference for assembly and annotation. The tRNA genes were annotated on ARAGORN (Laslett and Canback 2004).

The complete cp genome of *A. tutcheri* (GenBank accession number: MT580341) was 156,973 bp in length with the GC contents of 37.9%. It contains a pair of inverted repeats (IRs) of 26,753 bp, a small single copy region (SSC) of 18,111 bp and a large single copy region (LSC) of 85,356 bp. The genome encodes 132 unique genes, including 37 tRNA genes, eight rRNA genes and 87 protein-coding genes. Among these genes, 15 genes (*trnK*-*UUU*, *rps16*, *trnT*-*CGU*, *atpF*, *rpoC1*, *trnL*-*UAA*, *trnV*-*UAC*, *petB*, *petD*, *rpl16*, *rpl2*, *ndhB*, *trnE*-*UUC*, *trnA*-*UGC* and *ndhA*) have one intron and three genes (*clpP*, *rps12* and *ycf3*) have two introns.

Phylogenetic analysis was performed by using RAxML (Stamatakis 2006) with 1000 bootstrap replicates to identify the phylogenetic position of *A. tutcheri*. The matrix, includeing 32 whole cp genomes (30 species in *Acer* and two outgroups in *Dipteronia*), was aligned in MAFFT (Katoh and Standley 2013). The phylogenetic tree indicates that the monophyly of the genus *Acer* is well-supported (Figure 1). *Acer tutcheri* is closely related to *A. wilsonii* Rehd.

**Figure 1.**
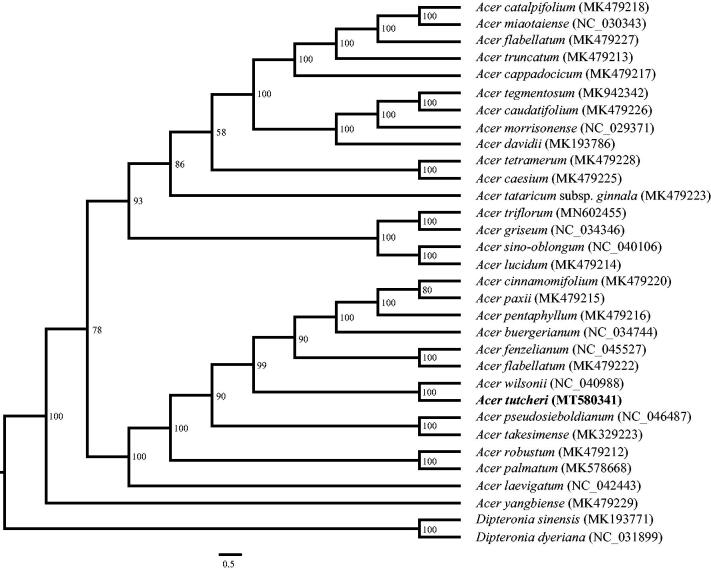
Maximum likelihood tree based on 32 complete chloroplast genomes of Acereae. *Dipteronia sinensis* and *D. dyeriana* were used as outgroups. Bootstrap support values are shown at the branches.

## Data Availability

The data that support the findings of this study are openly available in GenBank of NCBI at https://www.ncbi.nlm.nih.gov, reference number MT580341.
